# Angle-Retaining Chromaticity and Color Space: Invariants and Properties

**DOI:** 10.3390/jimaging8090232

**Published:** 2022-08-29

**Authors:** Marco Buzzelli

**Affiliations:** Department of Informatics, Systems and Communication, University of Milano—Bicocca, 20126 Milano, Italy; marco.buzzelli@unimib.it

**Keywords:** color space, chromaticity diagram, angular error, color invariants, explainability

## Abstract

The angle-retaining color space (ARC) and the corresponding chromaticity diagram encode information following a cylindrical color model. Their main property is that angular distances in RGB are mapped into Euclidean distances in the ARC chromatic components, making the color space suitable for data representation in the domain of color constancy. In this paper, we present an in-depth analysis of various properties of ARC: we document the variations in the numerical precisions of two alternative formulations of the ARC-to-RGB transformation and characterize how various perturbations in RGB impact the ARC representation. This was done empirically for the ARC diagram in a direct comparison against other commonly used chromaticity diagrams, and analytically for the ARC space with respect to its three components. We conclude by describing the color space in terms of perceptual uniformity, suggesting the need for new perceptual color metrics.

## 1. Introduction

A color space can be defined as “a geometric construct in which points that represent colors (or color stimuli) are arranged according to some principle” [1]. Such principles are typically driven by the application purpose of the color space itself. For example, the separation of an intensity-like component from the chromatic components is a key element in image compression, enhancement, and analysis. In the case of image compression, the JPEG algorithm and the corresponding JFIF file interchange format support and encourage using the Y’CbCr color space, so that the chromatic channels Cb and Cr can be encoded at a lower resolution, leveraging on the reduced sensitivity of the human eye to color details. The same principle is applied within the high-efficiency video coding (HEVC), also known as H.265. For image enhancement, Tang et al. [2] proposed a diffusion-based algorithm that decomposes the input image into its chromaticity and brightness components, which are subsequently processed using two distinct and dedicated approaches. Similarly, Vazquez-Corral et al. [3] approached image denoising with a color-sensitive decomposition of the input color information. Regarding image analysis, Li et al. [4] defined the quantized diagnostic counter-color pattern (QDCP): a novel rotation-invariant texture descriptor used in combination with local binary patterns [5] to identify and model chromatic similarity.

The separation of chromatic and intensity components was also proven to be useful in the visualization and analysis of information related to illuminant estimation and computational color constancy, with the definition of the angle-retaining color space [6] and angle-retaining chromaticity diagram [7] (called ARC space and ARC diagram for short). Computational color constancy is a discipline that aims to reduce the color cast of the dominant light source in a digital image, mimicking a similar mechanism observed in the human visual system. It is often addressed as a two-stage process: illuminant estimation and illuminant correction (also referred to as chromatic adaptation), which are evaluated against a ground truth illuminant. When such ground truth is provided in the form of a red–green–blue (RGB) triplet, the recovery angular error [8,9] is used to quantify the angular distance between the estimated illuminant $V=\left(v_{R},v_{G},v_{B}\right)$ and the ground truth illuminant $U=\left(u_{R},u_{G},u_{B}\right)$:

(1) $${\mathrm{err}}_{\mathrm{rec}}=\arccos\left(\frac{U\cdot V}{\left|U||V\right|}\right)=\arccos\left(\frac{\sum_{i}u_{i}v_{i}}{\sqrt{\sum_{i}u_{i}^{2}}\sqrt{\sum_{i}v_{i}^{2}}}\right)$$

Similarly, the reproduction angular error [10] is used to quantify the illuminant correction based on the same two elements:

(2) $${\mathrm{err}}_{\mathrm{rep}}=\arccos\left(\frac{\frac{U}{V}\cdot \left(1,1,1\right)}{|\frac{U}{V}|\sqrt{3}}\right)=\arccos\left(\frac{\sum_{i}\frac{u_{i}}{v_{i}}}{\sqrt{\sum_{i}\frac{u_{i}^{2}}{v_{i}^{2}}}\sqrt{3}}\right)$$

By representing illuminants as three-dimensional vectors and focusing on their angular distances, the recovery and reproduction errors effectively consider only the “difference” in illuminant chromaticity, disregarding the different intensities, which are not considered relevant for the chromatic characterization of light sources. This formulation is known to have a low correlation with human perception, as corroborated later on in Section 5, but it offers a straightforward tool to efficiently compute color differences.

It should be noted that computational color constancy, its evaluation, and related data visualization, are typically performed in a device-specific RAW-RGB color space [11] and derivative chromaticity diagrams. Such chromaticity diagrams are typically based on a normalization of the three RGB components, as in the case of the ratio [12,13], uv [11,14,15,16], and rg [16,17,18,19,20,21,22] diagrams, or on geometric transformations, such as the Maxwell triangle [23]. These will be presented in detail in Section 3. Alternative representations of color information involve physiologically-inspired color spaces, such as CIE XYZ [24] and its two-dimensional chromaticity counterpart known as CIE xy, and its derivations CIE L*u*v* and CIE L*a*b* [25], which are aimed at better perceptual uniformity and are often used in industrial applications for faithful color reproduction, or even the Munsell color order system [26]. Given our focus on computational color constancy in digital images, we compare ARC against RGB-derived chromaticity diagrams. In Section 5, we will also refer to the Munsell system to provide a perceptual characterization of the presented ARC space.

Angular-based error metrics are the established de facto standards in computational color constancy. The ARC diagram, and by extension the ARC space, was designed to characterize the input RGB information by representing the chromatic components so that angular distances in RGB are retained as Euclidean distances. As a result, visual inspection of color constancy data (such as error distributions) will not be biased by distortions in the underlying data representation. For example, when plotting reproduction errors in the ARC diagram, it is possible to have a direct impression of the error distribution for different chromaticities, proportional to their actual roles in the overall angle-based error evaluation. This cannot be achieved with other diagrams that introduce various distortions, as shown in [7]. Additionally, ARC allows performing analyses and operations that were originally designed for Euclidean spaces, in the context of angular distances. For example, when describing the distribution of illuminants in video sequences, Buzzelli et al. [27] used a metric of two-dimensional scatteredness called standard distance [28] on ARC diagram coordinates, effectively embedding angular distances in this characterization. This would not have been possible by directly computing the standard distance in RGB coordinates, as Euclidean distances in a 3D space are not well correlated with angular distances. In [7], the diagram was originally defined and applied to the comparison of color constancy datasets and methods. In [6], its expansion as a full-fledged color space was explored in terms of its potential application to the fields of image enhancement and classification based on texture analysis. Adding to the previous research, in this paper, we provide a novel formal characterization of the ARC chromaticity diagram and color space. Specifically:

1. We compare two alternative formulations of the conversion from ARC to RGB, based on different geometric interpretations: a first formulation described in the original introduction of the ARC space [6], and a second formulation derived from a work by Chen et al. [29] on the definition of a spherical color space. Specifically for this paper, we characterize the two formulations in terms of precision in numerical representation.
2. We characterize how small perturbations in the original RGB triplets map as changes in various chromaticity diagrams. A similar analysis was originally conducted in [7], providing a shape description of the local distortions introduced with chromaticity representations. In contrast, here we instead describe the intensity of such deformations, formulated with a novel approach. This information is both presented qualitatively with intuitive representations, as well as quantified numerically.
3. We present an analytical study on the invariance of each component of the ARC space to various perturbations in the input RGB triplets as related to different changes in environmental illumination conditions. This analysis borrows existing definitions of light transformations [30], and provides an entirely original mathematical derivation.
4. We explore the perceptual properties of the ARC space by referring to the standard Munsell hue data, and propose a direction for improvement in future works. Previous works related to ARC did not provide any study or insight into its relationship with physiological data, which can be considered important for color reproduction applications.

## 2. Angle-Retaining Chromaticity and Color Space

### 2.1. RGB-to-ARC Transformation

Buzzelli et al. [7] defined the angle retaining chromaticity diagram as a two-dimensional (2D) representation of color information, disregarding the intensity. A complete three-dimensional (3D) angle-retaining color space is then defined in [6]. The resulting three components are:

(3) $$\alpha_{A}=\arctan2\left(\sqrt{3}\left(G-B\right),2R-G-B\right)$$

(4) $$\alpha_{R}=\arccos\left(\frac{R+G+B}{\sqrt{3}\sqrt{R^{2}+G^{2}+B^{2}}}\right)$$

(5) $$\alpha_{Z}=\sqrt{R^{2}+G^{2}+B^{2}}$$

**Figure 1:**
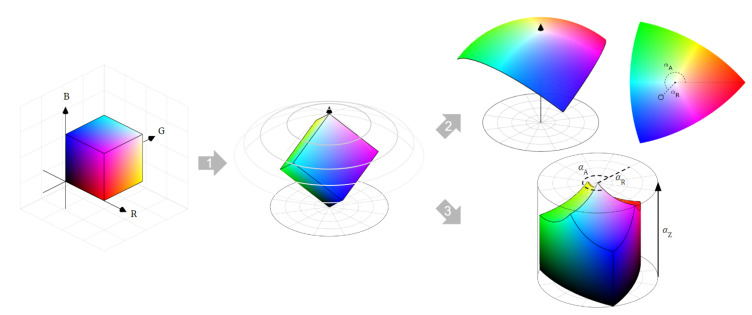
Geometric interpretation of the RGB-to-ARC conversion. Step 1 applies a rotation and a change in the coordinate system. Step 2 provides the transformation into the 2D ARC diagram. Step 3 provides the transformation into the 3D ARC space.

The transformation from RGB to ARC space can be interpreted geometrically by referring to Figure 1, in terms of operations applied to the RGB gamut cube:

1. The RGB cube is rotated so that the neutral gray axis becomes the new vertical axis, and so that the pure green vertex is positioned in the 3D octant of the resulting coordinate system having negative *x*, and positive *y* and *z*. The representation is also brought from Cartesian coordinates into polar coordinates (e.g., ρ,θ,ϕ).
2. For the 2D ARC diagram, the radial distance ρ is intentionally dropped, to discard the intensity information. The authors in [7] show that this operation corresponds to expanding all RGB points to the surface of a sphere with the center in the origin and flattening such a sphere by following an equidistant projection, which preserves the great-circle distances with respect to the point of neutral grays. This guarantees the main properties that angular distances in RGB are retained as Euclidean distances in ARC.
3. For the 3D ARC space, the spherical polar coordinates are reinterpreted as cylindrical polar coordinates, and if necessary brought into Cartesian coordinates. As shown by the authors [6], this guarantees that the angle-retaining property of ARC is maintained for each “horizontal slice” of the 3D space (i.e., by ignoring the third dimension when computing Euclidean distances).

**Figure 2:**
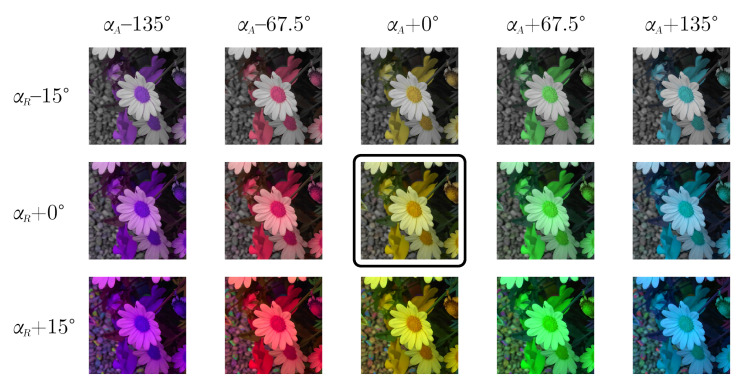
Visualization of how modifying ARC chromatic components $\alpha_{A}$ and $\alpha_{R}$ impacts the reconstructed RGB image. Original image in the middle center.

Figure 2 depicts a chamomile flower (in the middle center), modified by shifting its $\alpha_{A}$ and $\alpha_{R}$ ARC chromaticity values. In this particular example, reducing $\alpha_{R}$ has the effect of desaturating the yellow petals to the point of being white, while changing $\alpha_{A}$ introduces an overall hue shift.

### 2.2. ARC-to-RGB Transformation

Inverting the transformation from 3D ARC to RGB can be achieved with two alternative formulations, each presenting a unique intuition about the underlying geometric transformations.

Buzzelli et al. [6] derived the inversion by describing the 3D ARC point as the intersection between:

- A half-plane in the RGB space that hinges on the neutral axis, with an orientation depending on the value of $\alpha_{A}$.
- An infinite cone with its vertex in the RGB origin, and its axis corresponding to the neutral axis. The aperture of this cone is directly related to the value of $\alpha_{R}$.
- A sphere having its center in the RGB origin, and radius equivalent to $\alpha_{Z}$.

**Figure 3:**
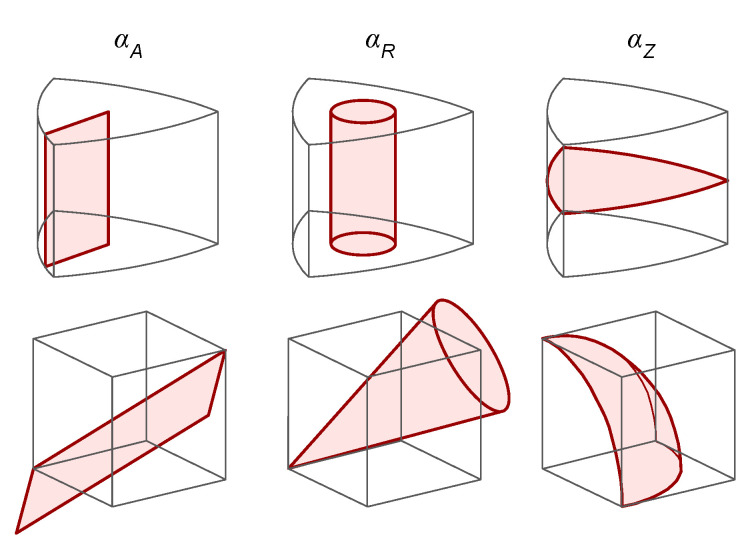
Correspondence between ARC components $\alpha_{A}$, $\alpha_{R}$, $\alpha_{Z}$, and the surface in the ARC and RGB space that each component identifies.

The correspondence between the aforementioned geometric surfaces and the ARC components are visualized in Figure 3.

This formulation leads to expressing *R*, *G*, and *B* as:

(6) $$R=\frac{k_{R}}{\sqrt{k_{R}^{2}+k_{G}^{2}+k_{B}^{2}}}\alpha_{Z}$$

(7) $$G=\frac{k_{G}}{\sqrt{k_{R}^{2}+k_{G}^{2}+k_{B}^{2}}}\alpha_{Z}$$

(8) $$B=\frac{k_{B}}{\sqrt{k_{R}^{2}+k_{G}^{2}+k_{B}^{2}}}\alpha_{Z},$$

where $k_{R}$, $k_{G}$, and $k_{B}$ describe the Cartesian relationship between $\alpha_{A}$ and $\alpha_{R}$:

(9) $$k_{R}=\left|3sgn\left(\alpha_{A}\right)sgn\left(c\right)\sqrt{\left(c^{2}-c+1\right)d}+\left(c^{2}-c-2\right)d-\left(c^{2}-c+1\right)\right|$$

(10) $$k_{G}=\left|\left(c^{2}+2c+1\right)d-\left(c^{2}-c+1\right)\right|$$

(11) $$k_{B}=\left|3sgn\left(\alpha_{A}\right)\sqrt{c^{2}\left(c^{2}-c+1\right)d}-\left(2c^{2}+c-1\right)d-\left(c^{2}-c+1\right)\right|.$$

These expressions are, in turn, based on the half-plane and cone parameters *c* and *d*:

(12) $$c=\frac{2\sqrt{3}}{\sqrt{3}-3\cot\left(\alpha_{A}\right)}$$

(13) $$d=\frac{\tan{\left(\alpha_{R}\right)}^{2}}{2}$$

This formulation, based on the intersection of three independent surfaces, provides a visual interpretation that can be useful in studying invariant properties (transformations of the color triplet that are restricted to either of these surfaces). Such a study on color transformation invariance is presented in Section 4.

A second (alternative) inversion can be derived based on the work by Chen et al. [29] on the definition of a spherical color space. Here, the inverse transformation can be seen as direct backtracking of the geometric passages illustrated in Figure 1:

1. Transformation from the polar coordinates to Cartesian coordinates.
2. Inverse rotation of the three axes.

This yields:

(14) $$R=\frac{\sqrt{6}}{3}\cos\alpha_{A}\sin\alpha_{R}\alpha_{Z}+\frac{1}{\sqrt{3}}\cos\alpha_{R}\alpha_{Z}$$

(15) $$G=-\frac{1}{\sqrt{6}}\cos\alpha_{A}\sin\alpha_{R}\alpha_{Z}+\frac{1}{\sqrt{2}}\sin\alpha_{A}\sin\alpha_{R}\alpha_{Z}+\frac{1}{\sqrt{3}}\cos\alpha_{R}\alpha_{Z}$$

(16) $$B=-\frac{1}{\sqrt{6}}\cos\alpha_{A}\sin\alpha_{R}\alpha_{Z}-\frac{1}{\sqrt{2}}\sin\alpha_{A}\sin\alpha_{R}\alpha_{Z}+\frac{1}{\sqrt{3}}\cos\alpha_{R}\alpha_{Z}$$

**Figure 4:**
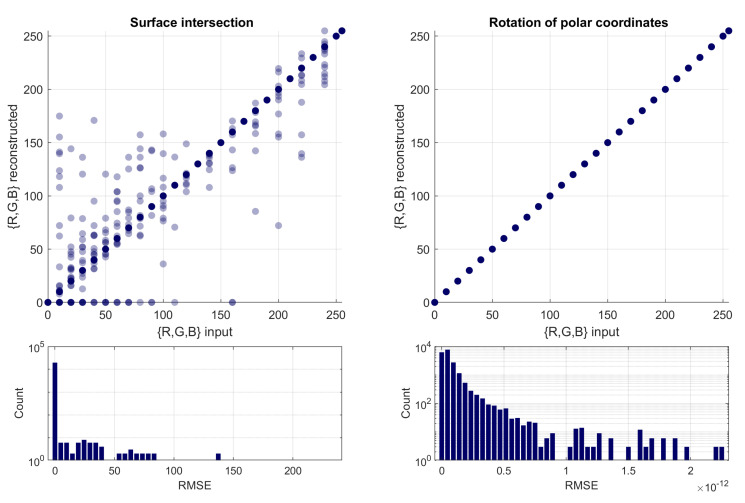
Comparison between ARC-to-RGB inversion based on surface intersection [6] (**left**), and the rotation of the polar coordinates [29] (**right**). The top row shows the correlation between the input and reconstruction, measured in terms of the Pearson linear coefficient, respectively, as 0.9994 and 1.0000. The bottom row shows the details of the error distribution: note the difference in the horizontal scale.

In order to verify the effectiveness, in terms of numerical accuracy, of the two approaches, we sampled a set of random RGB points, converted them into ARC space following Equations (3)–(5), and then inverted the resulting point back into RGB by following either the formulation based on surface intersection [6], or the one based on the rotation of polar coordinates [29]. The input and reconstructed RGB points should, ideally, be identical. The results are reported in Figure 4, visualizing the correlation between input and reconstructed values (each color component taken individually), as well as their error distributions in terms of root mean square error (RMSE). By quantifying the two correlations in terms of Pearson linear coefficient, we obtain, respectively, 0.9994 and 1.0000. Both suggest a robust numerical representation; however, it can be observed how the formulation based on the surface intersection introduces sparse occurrences of large errors, which can be traced to extremely saturated input colors. Although such colors are rarely encountered in the application domain of computational color constancy, for the sake of generality, the formulation based on the rotation of the polar coordinates appears superior, and it is now adopted in the official code implementation of the ARC space [31].

## 3. Properties of the 2D ARC Diagram

**Figure 5:**
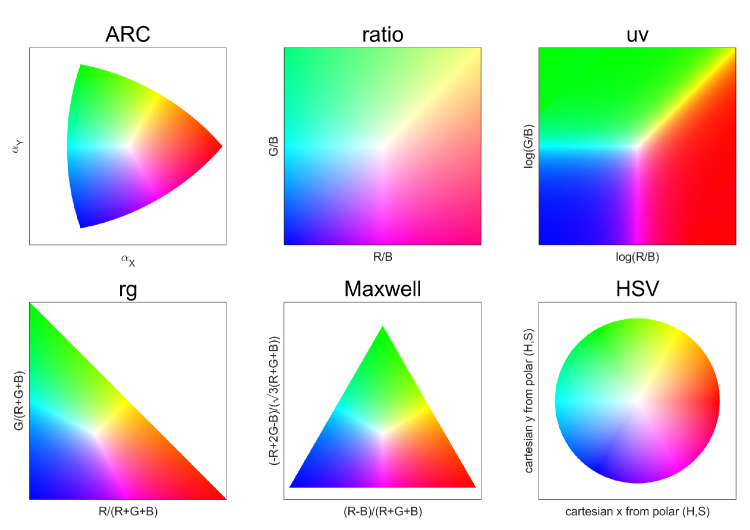
RGB gamut as displayed in different chromaticity diagrams.

We present an analysis of the properties related to the 2D ARC diagram, by selecting—as a benchmark for comparison—a number of alternative chromaticity diagrams, which are typically used as visualization aids or as representation spaces for color-related image processing. These are visualized in Figure 5, displaying the equivalent of the RGB gamut in each diagram.

1. “ARC”’s axes are here labeled as $\alpha_{X}$ and $\alpha_{Y}$ in reference to the Cartesian coordinate version of $\alpha_{A}$ and $\alpha_{R}$. The ARC diagram has been used for pure data visualization purposes [32,33] and data representation purposes, to exploit angular distances in operations originally designed for Euclidean distances [27,34].
2. The “ratio” is obtained by normalizing two color components for a third one (in the example, RG and BG). This representation was used to represent chromaticity histograms for illuminant estimation [12], and to present the INTEL-TUT dataset for camera-invariant color constancy [13].
3. “uv” is computed as the logarithm of the ratios from the previous diagram, as a measure to partially constrain its domain. It was introduced in [14], and used to encode color information in various color constancy methods [11,15,16], and again for data presentation [35].
4. “rg“ is obtained by dividing two color components (R and G in the example) by the sum of all three. It is often used due to its simplicity in data visualization [16,17,18,19,20] as well as representation for illuminant estimation [21,22].
5. “Maxwell” is computed by projecting the three components onto a plane perpendicular to the neutral axis. It is named in reference to the pioneering works on color by James Clerk Maxwell [23].
6. “HSV” refers to the traditional hue–saturation–value color space, from which we consider the chromatic components hue and saturation. Although not typically used in the domain of color constancy, we chose to include it for comparison due to its conceptual similarity to ARC.

**Figure 6:**
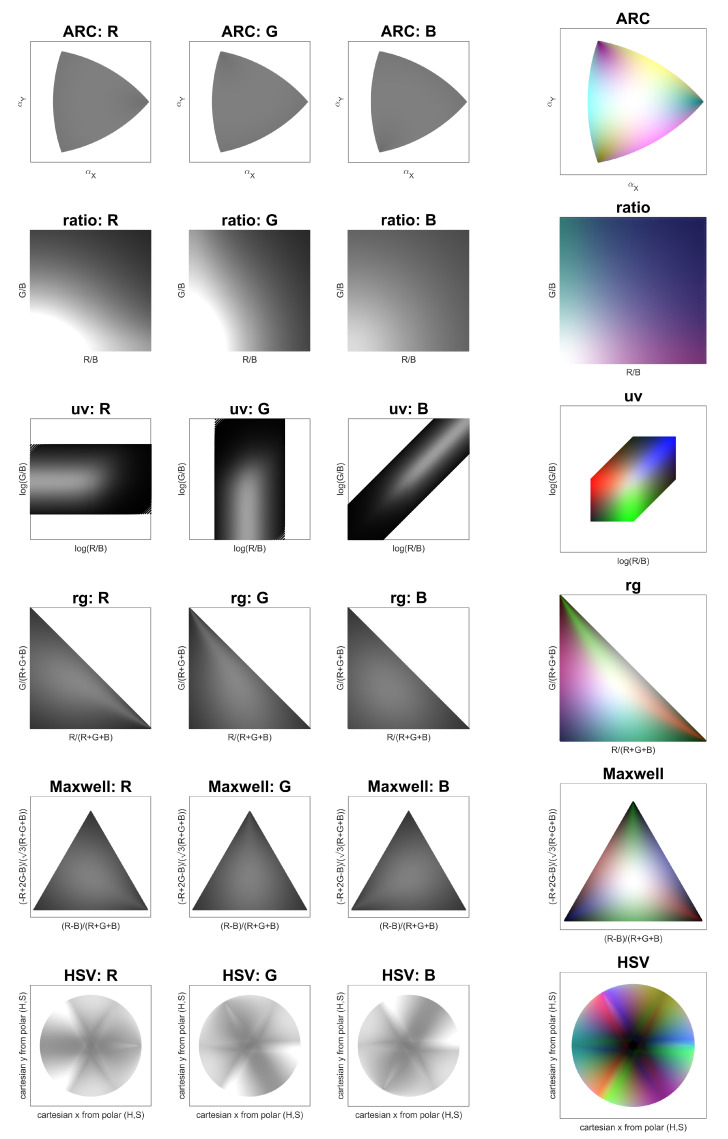
Visual representation of how perturbations on various RGB points map as changes in different chromaticity diagrams. The first three columns present three distortions independently (rotation of R, G, and B), for which flat values close to gray indicate better stability. The last column combines the three pieces of information for a qualitative evaluation of the strongest color distortions.

Specifically, we are interested in characterizing how small perturbations in the original RGB point, which could be produced by arbitrary physical phenomena impacting the starting illuminant, map as changes in a given chromaticity diagram. Ideally, variations of the same intensity in different areas of the RGB cube should produce the same effect in terms of chromaticity changes. This property would guarantee the absence of distortions in data representation as introduced by the chosen chromaticity diagram. However, it should be noted that a perfect match is not possible due to the implied dimensionality reduction from three to two components, as discussed in [7]. The results are illustrated in Figure 6.

The relationship between RGB and chromaticity distortion can be formulated as:

(17) $${\mathrm{dist}}_{\mathrm{diag},\{r,g,b\}}\left(G,G,B\right)=\frac{{\mathrm{err}}_{\mathrm{rec}}\left(\left[R\;G\;B\right]\cdot M_{\left\{r,g,b\right\}}\left(+\epsilon \right),\left[R\;G\;B\right]\cdot M_{\left\{r,g,b\right\}}\left(-\epsilon \right)\right)}{\mathrm{eud}\left(\mathrm{diag}\left(\left[R\;G\;B\right]\cdot M_{\left\{r,g,b\right\}}\left(+\epsilon \right)\right),\mathrm{diag}\left(\left[R\;G\;B\right]\cdot M_{\left\{r,g,b\right\}}\left(-\epsilon \right)\right)\right)},$$

where diag identifies the chromaticity diagram and the multiplication of the input point [RGB] by matrices $M_{\left\{r,g,b\right\}}$ realizes a rotation around either color axis, according to:

(18) $$M_{r}\left(\theta \right)=\left[\begin{array}{ccc} 1 & 0 & 0\\ 0 & \cos\theta & \sin\theta \\ 0 & -\sin\theta & \cos\theta \end{array}\right],$$

(19) $$M_{g}\left(\theta \right)=\left[\begin{array}{ccc} \cos\theta & 0 & -\sin\theta \\ 0 & 1 & 0\\ \sin\theta & 0 & \cos\theta \end{array}\right],$$

(20) $$M_{b}\left(\theta \right)=\left[\begin{array}{ccc} \cos\theta & \sin\theta & 0\\ -\sin\theta & \cos\theta & 0\\ 0 & 0 & 1 \end{array}\right].$$

The numerator in Equation (17) computes the angular error between the two RGB points following Equation (1), whereas the denominator measures the Euclidean distance between the two resulting 2D chromaticities:

(21) $$\mathrm{eud}\left(U,V\right)=\sqrt{\sum_{i}{\left(u_{i}-v_{i}\right)}^{2}}.$$

In Figure 6, we visually illustrate the application of Equation (17) to all RGB points lying on the outer shell of the RGB cube. The group of three columns on the left shows the effect of distortion (matrix multiplication) on each individual R, G, and B channel. For visualization, all values are normalized (divided) with respect to the following factor:

(22) $${\mathrm{norm}}_{\mathrm{diag},\{r,g,b\}}=\frac{{\mathrm{dist}}_{\mathrm{diag},\{r,g,b\}}\left(1,1,1\right)}{2}$$

This operation has two effects:

1. The distortion of each diagram is normalized for its distortion level in the case of a neutral gray, in order to remove a global bias related to different orders of magnitude.
2. The normalization allows observing, at a glance, the differences in distortion impacts across different diagrams.

In this sense, a strong distortion is visualized with values tending towards black or white, whereas gray indicates little or no distortion. For example, while ARC is reasonably flat, the ratio diagram in the immediate next row shows a strong distortion towards the bottom left corner, concerning the impact of R and G variations. The same information is quantified and reported in Table 1 in terms of the standard deviation of each distribution, for which a lower value implies less variation in the distortion. Reflecting what can be visually appreciated, the ARC diagram presents the lowest variability, followed by the Maxwell triangle and the rg diagram. The largest variation in distortion is introduced instead by the ratio chromaticity diagram, where the division by one color component introduces a strong non-linear dependence for that component itself.

The last column of Figure 6 presents once again a visualization of Equation (17), this time normalized for the min–max of each channel–diagram pair, with all three channels combined into one. In this case, the image is supposed to qualitatively convey the information about the type of chromatic distortions, regardless of their magnitude. For example, the uv and Maxwell diagrams introduce distortions directly correlated to the input red, green, and blue variations. Conversely, all other diagrams introduce distortions that result from a combination of the modified axes, such as cyan magenta and yellow.

## 4. Properties of 3D ARC Space

We investigated properties related to the 3D ARC space. Specifically, given a pair $\left(\alpha_{*},f\right)$, defining an ARC component $\alpha_{*}$, and a transformation *f* on the input RGB point, we intend to determine whether the following equation holds:

(23) $$ARC_{\alpha_{*}}\left([R\;G\;B]\right)=ARC_{\alpha_{*}}\left(f\left([R\;G\;B]\right)\right)$$

In geometric terms, this means verifying whether a given transformation of the input RGB triplet introduces a change that keeps the point, respectively:

- On the surface of the half-plane in the RGB space, whose equation is defined by $\alpha_{A}$.
- On the surface of the infinite cone in the RGB space, whose equation is defined by $\alpha_{R}$.
- On the surface of the sphere of the RGB space, whose equation is defined by $\alpha_{Z}$.

We refer the reader to Section 2.2 and Figure 3 for the correspondence between ARC components and surfaces. We consider (as transformations (*f*)) the light variations described by van de Sande et al. [30], as these can be traced back to specific variations in the lighting conditions. Ideally, if we know how individual variations in the environment lighting affect each component of the data representation, we could define descriptors and formulate analyses with known properties of invariance. Combinations of ARC components and transformations are reported in Table 2.

The definition of *Conditional invariance* is here formulated as: “there exist non-trivial solutions that involve a mutual compensation of simultaneous distortion elements”. For example, with respect to the “Light intensity change and shift” transformation described later on (where the input RGB triplet is multiplied by a scalar *a* and then summed by offset $o_{1}$) it is possible to define a value of $o_{1}$ as a function of R,G,B, and *a*, such that it compensates the effect of *a* in the intensity component $\alpha_{Z}$. By non-trivial solution, we mean that we exclude scalar a=1 and offset $o_{1}=0$.

In the following, we provide an overview of each transformation presented in Table 2. We refer the reader to Appendix A for the derivation of the stated invariance of each component/transformation pair.

### 4.1. Light Intensity Change

Described by the expression:

(24) $$\left(\begin{array}{ccc} a & 0 & 0\\ 0 & a & 0\\ 0 & 0 & a \end{array}\right)\left(\begin{array}{c} R\\ G\\ B \end{array}\right),$$

it can be associated with the effects of achromatic shadows and shading phenomena, or, in other terms, lighting geometry changes [30]. By construction, the chromatic components of ARC ($\alpha_{A}$ and $\alpha_{R}$) are invariant to this transformation, which corresponds to a pixel value being made brighter or darker. For the same motivation, the intensity component $\alpha_{Z}$ cannot be invariant to this transformation.

### 4.2. Light Intensity Shift

Described by the expression:

(25) $$\left(\begin{array}{c} R\\ G\\ B \end{array}\right)+\left(\begin{array}{c} o_{1}\\ o_{1}\\ o_{1} \end{array}\right),$$

this transformation is defined as capturing the behavior of diffuse lighting, object highlights, mutual inter-reflections between objects, as well as the impact of infrared sensitivity observed in certain imaging sensors [30]. The hue-like component $\alpha_{A}$ can be shown to be fully invariant to this type of transformation. Conversely, the saturation component $\alpha_{R}$ and intensity component $\alpha_{Z}$ only admit a conditional invariance that would require negative RGB values, effectively resulting in being non-invariant.

### 4.3. Light Intensity Change and Shift

Described by the transformation:

(26) $$\left(\begin{array}{ccc} a & 0 & 0\\ 0 & a & 0\\ 0 & 0 & a \end{array}\right)\left(\begin{array}{c} R\\ G\\ B \end{array}\right)+\left(\begin{array}{c} o_{1}\\ o_{1}\\ o_{1} \end{array}\right),$$

this is a combination of the transformations described in Equations (24) and (25). In general, when both sub-transformations produce an invariant, so will their combination, whereas the lack of invariance from either sub-transformation implies the same non-invariance for their combination. However, by referring to the provided definition of the conditional invariance, it can be shown that $\alpha_{Z}$ can be preserved under a “light intensity change and shift” transformation, as long as the offset $o_{1}$ cancels out the effect of scalar *a*, which can be achieved following this relationship:

(27) $$\begin{array}{c} o_{1}=\frac{1}{3}\left(\pm \sqrt{\left(3-2a^{2}\right)B^{2}+2a^{2}B\left(G+R\right)+\left(3-2a^{2}\right)G^{2}+2a^{2}GR+\left(3-2a^{2}\right)R^{2}}-a\left(B+G+R\right)\right) \end{array}$$

### 4.4. Light Color Change

Described by the transformation:

(28) $$\left(\begin{array}{ccc} a & 0 & 0\\ 0 & b & 0\\ 0 & 0 & c \end{array}\right)\left(\begin{array}{c} R\\ G\\ B \end{array}\right),$$

this is equivalent to the von Kries-like transform [36] that is often used in the domain of computational color constancy to model the illuminant correction phase. Although known to be suboptimal and unable to fully handle metameric effects [37], the von Kries transform is commonly adopted due to its simple formulation. In its general form, the transformation from Equation (28) prevents invariance for all ARC components. However, conditional invariance can be achieved by formulating scalar *c* as a function of the other scalars, *a* and *b*. Specifically, conditional invariance for $\alpha_{A}$ is given by:

(29) $$c=\frac{-\frac{2\sqrt{3}aR\left(B-G\right)}{B+G-2R}+\frac{\sqrt{3}bG\left(B-G\right)}{B+G-2R}+\sqrt{3}bG}{\sqrt{3}B-\frac{\sqrt{3}B\left(B-G\right)}{B+G-2R}}$$

Conditional invariance for $\alpha_{R}$ is given by:

(30) $$c=\frac{\pm \sqrt{B^{2}{\left(-2aR-2bG\right)}^{2}-4B^{2}\left(\frac{{\left(B+G+R\right)}^{2}}{B^{2}+G^{2}+R^{2}}-1\right)\left(\frac{a^{2}R^{2}{\left(B+G+R\right)}^{2}}{B^{2}+G^{2}+R^{2}}-a^{2}R^{2}-2abGR+\frac{b^{2}G^{2}{\left(B+G+R\right)}^{2}}{B^{2}+G^{2}+R^{2}}-b^{2}G^{2}\right)}-B\left(-2aR-2bG\right)}{2B^{2}\left(\frac{{\left(B+G+R\right)}^{2}}{B^{2}+G^{2}+R^{2}}-1\right)}$$

Finally, conditional invariance for $\alpha_{Z}$ is given by:

(31) $$c=\frac{\sqrt{-a^{2}R^{2}-b^{2}G^{2}+B^{2}+G^{2}+R^{2}}}{B}$$

### 4.5. Light Color Change and Shift

Described by the transformation:

(32) $$\left(\begin{array}{ccc} a & 0 & 0\\ 0 & b & 0\\ 0 & 0 & c \end{array}\right)\left(\begin{array}{c} R\\ G\\ B \end{array}\right)+\left(\begin{array}{c} o_{1}\\ o_{2}\\ o_{3} \end{array}\right),$$

this is the most general transformation considered in this paper, combining the von Kries-like transform from Equation (28) with a colored offset. Geometrically, it is equivalent to an affine transformation applied to the RGB coordinates. The transformation inherits the conditional invariant properties relative to Equation (28), in addition to specific constraints for the offsets $o_{1}$, $o_{2}$, and $o_{3}$. Conditional invariance for $\alpha_{A}$ is given by:

(33) $$o_{3}=\frac{-Bo_{1}+Bo_{2}+Go_{1}-o_{2}R}{G-R}$$

Conditional invariance for $\alpha_{z}$ is given by:

(34) $$o_{3}=\pm \sqrt{B^{2}-o_{2}\left(2G+o_{2}\right)-o_{1}^{2}-2o_{1}R}-B$$

Given its simple formulation, conditional invariance for $\alpha_{Z}$ can also be directly expressed without decomposition between scalars *a*, *b*, *c*, and offsets $o_{1}$, $o_{2}$, $o_{3}$ as:

(35) $$o_{3}=\pm \sqrt{-a^{2}R^{2}-2ao_{1}R-b^{2}G^{2}-2bGo_{2}+B^{2}+G^{2}-o_{1}^{2}-o_{2}^{2}+R^{2}}-Bc$$

As a general comment, while the invariance properties related to “Light intensity change” could be considered trivial, resulting from construction, the “Light intensity shift” and “Light intensity change and shift” invariance for $\alpha_{A}$ are important pieces of information to characterize the response of the ARC representation to these types of changes in the lighting conditions. Understanding the mechanics of how light influences data representation, and its subsequent processing in, for example, color constancy, constitutes a step towards the concept of explainable artificial intelligence, where each building block is thoroughly characterized instead of being considered a black box. In terms of conditional invariance, the emerged properties open the possibility for specific illumination conditions to not impact one or more ARC components. What is described here only in terms of mathematical relationships could be further analyzed in the future with the aid of visualization techniques in order to provide intuitive interpretations and practical applications.

## 5. ARC Properties Related to Human Perception

We conclude with a preliminary investigation of the properties of the ARC space and diagram relative to human perception. We approach this analysis by considering hue lines from the Munsell Color Order System [26], and observing how they appear in ARC.

We consider the “renotation” colors from a large-scale visual experiment performed in 1943 with observers across several continents [38]. These include patches annotated in terms of Munsell hue (40 possible hues), Munsell value (values from 1 to 9), and Munsell chroma (from 2 to 26), for a total of 1625 patches. These are converted into CIE xyY chromaticity coordinates using illuminant C and the CIE 1931 2-degree observer and eventually brought into sRGB assuming a D65 illuminant [39]. Finally, the sRGB coordinates are converted into ARC following Equations (3)–(5).

**Figure 7:**
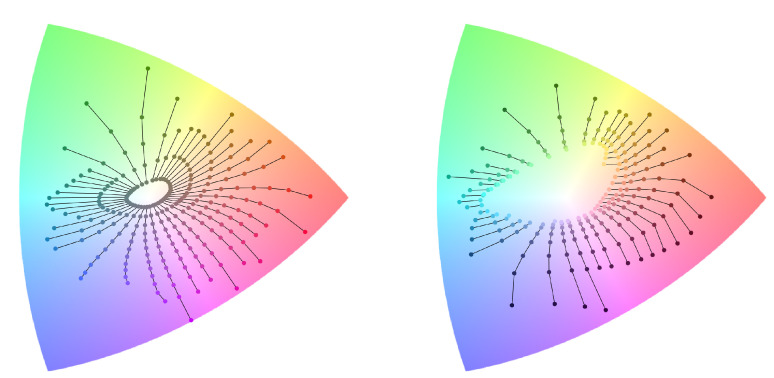
Visualization of Munsell patches converted from sRGB to ARC diagram, grouped by the Munsell hue. On the left: patches having Munsell value = 5. On the right: patches having Munsell chroma = 8. The underlying colors of the ARC diagram have been reduced in contrast for better visualization of the Munsell datapoints.

We define two experiments, as shown in Figure 7:

1. We select all patches with intermediate Munsell value = 5; therefore, varying the Munsell hue and Munsell chroma, and plotting them in the ARC diagram, connecting patches relative to the same Munsell hue group. The resulting plot, shown in Figure 7 (left) with 40 hues radiating from the center, is not composed of straight lines, especially for highly-saturated purple, red, and lime areas. This implies that moving in the direction defined by the $\alpha_{R}$ component does not guarantee perceived hue invariance.
2. We select all patches with intermediate Munsell chroma = 8; therefore, varying the Munsell hue and Munsell value, and once again plotting them in ARC and grouping the points by the Munsell hue. The result, shown in Figure 7 (right), is equivalent to plotting the information in the 3D arc space and then observing the data from the “top view”, i.e., neglecting the intensity component. In addition to the already commented upon the effect of hue instability, it can be observed that the Munsell value does not correspond to the ARC intensity component: each Munsell hue group in fact appears as radiating from the center, instead of being composed of overlapping points.

Generally speaking, the observed mismatch between Munsell coordinates and ARC components implies that the ARC representation does not natively offer properties of perceptual uniformity when used to encode sRGB data. Given the tight relationship between the ARC representation and angular errors, this experiment also reflects the underlying gap between simple angle-based metrics and human-perceived differences. Therefore, we hypothesize that it is not possible to enforce perceptual uniformity while also preserving the angle-retaining properties that characterize ARC. We argue that there is an advantage in separating the objective and subjective aspects of any evaluation, including the evaluation of the color constancy itself. Disregarding the intensity component is an objective requirement, embedded in the very definition of color constancy, concerned with a purely chromatic characterization of the light sources. The actual sensitivity to different chromaticities (such as sensitivity to the yellow–blue axis as opposed to the green–magenta axis) is instead subjective in nature: it might vary due to individual differences [40], and it should take into account external factors, such as the scene content and the conditions under which the image is viewed [8]. For this reason, we suggest that additional properties be embedded by defining custom metrics, such as CIE ΔE for the CIE L*a*b* color space. This would allow maintaining the main angle-retaining property of ARC, while including further characteristics with distance metrics that can be adapted and changed based on each use case.

## 6. Conclusions

We presented an in-depth analysis of the ARC space and ARC diagram under different points of view.

The numerical precisions of two ARC-to-RGB inversion formulas were compared, informing the most-appropriate adoption in the official ARC implementation. Different chromaticity diagrams were compared in terms of their robustness to perturbations of the input RGB values, with ARC resulting in the most stable solution, followed by Maxwell’s triangle and rg chromaticity. The invariances of the three ARC components, with respect to various changes in lighting conditions, were analytically documented. With respect to conditional invariance, these properties were found to describe only very specific conditions for which invariance was met: further analysis of these constraints with the aid of visualization techniques could potentially highlight their practical application. The perceptual uniformity of ARC was investigated by referring to Munsell hue lines, suggesting a relatively low correlation with human perception. This characteristic could be addressed in the future by embedding custom distance metrics in the ARC color space, in order to still preserve its defining angle-retaining characteristics when relying on Euclidean distances.

## Figures and Tables

**Table 1:** Standard deviation of the ratio between distortion in RGB space and distortion in chromaticity, for different chromaticity diagrams and considering different distortions (rotations around the R, G, or B axis). A lower value is better. The average column is supported by ranking information.

Chromaticity Diagram	St. Dev. (R)	St. Dev. (G)	St. Dev. (B)	St. Dev. (Average)
ARC	0.0103	0.0103	0.0103	0.0103${}_{\left(1\right)}$
ratio	0.3945	0.3945	0.2245	0.3378${}_{\left(6\right)}$
uv	0.1630	0.1630	0.1658	0.1639${}_{\left(5\right)}$
rg	0.0769	0.0769	0.0556	0.0698${}_{\left(3\right)}$
Maxwell	0.0609	0.0609	0.0609	0.0609${}_{\left(2\right)}$
HS (HSV)	0.1193	0.1193	0.1193	0.1193${}_{\left(4\right)}$

**Table 2:** Invariant properties of each ARC component $\alpha_{A}$, $\alpha_{R}$, $\alpha_{Z}$, with respect to various transformations in light conditions.

ARC Component	Light Intensity Change $\left(\begin{array}{ccc} a & 0 & 0\\ 0 & a & 0\\ 0 & 0 & a \end{array}\right)\left(\begin{array}{c} R\\ G\\ B \end{array}\right)$	Light Intensity Shift $\left(\begin{array}{c} R\\ G\\ B \end{array}\right)+\left(\begin{array}{c} o_{1}\\ o_{1}\\ o_{1} \end{array}\right)$	Light Intensity Change and Shift $\left(\begin{array}{ccc} a & 0 & 0\\ 0 & a & 0\\ 0 & 0 & a \end{array}\right)\left(\begin{array}{c} R\\ G\\ B \end{array}\right)+\left(\begin{array}{c} o_{1}\\ o_{1}\\ o_{1} \end{array}\right)$	Light Color Change $\left(\begin{array}{ccc} a & 0 & 0\\ 0 & b & 0\\ 0 & 0 & c \end{array}\right)\left(\begin{array}{c} R\\ G\\ B \end{array}\right)$	Light Color Change and Shift $\left(\begin{array}{ccc} a & 0 & 0\\ 0 & b & 0\\ 0 & 0 & c \end{array}\right)\left(\begin{array}{c} R\\ G\\ B \end{array}\right)+\left(\begin{array}{c} o_{1}\\ o_{2}\\ o_{3} \end{array}\right)$
$\alpha_{A}$ (Hue-like)	Invariant	Invariant	Invariant	Conditional	Conditional
$\alpha_{R}$ (Saturation-like)	Invariant	Not invariant	Not invariant	Conditional	Conditional
$\alpha_{Z}$ (Intensity-like)	Not invariant	Not invariant	Conditional	Conditional	Conditional

## Data Availability

Not applicable.

## Appendix A. Properties of 3D ARC Space—Derivation

In the following, we provide a demonstration for the invariance and conditional-invariance properties of the ARC space with respect to variations in the light conditions. These are obtained by finding the solutions to the following equation:

(A1) $$ARC_{\alpha_{*}}\left([R\;G\;B]\right)=ARC_{\alpha_{*}}\left(f\left([R\;G\;B]\right)\right)$$

for all three ARC components $\alpha_{*}$ and five different transformations *f*.

### Appendix A.1. Light Intensity Change

Described by the expression:

(A2) $$\left(\begin{array}{ccc} a & 0 & 0\\ 0 & a & 0\\ 0 & 0 & a \end{array}\right)\left(\begin{array}{c} R\\ G\\ B \end{array}\right)$$

*$\alpha_{A}$ component:*

(A3)
$$\arctan2\left(\sqrt{3}\left(G-B\right),2R-G-B\right)=\arctan2\left(\sqrt{3}\left(aG-aB\right),2aR-aG-aB\right)$$

Scalar *a* can be collected in both arguments from the right part of the equation, leading to:

(A4) $$\arctan2\left(\sqrt{3}\left(G-B\right),2R-G-B\right)=\arctan2\left(a\sqrt{3}\left(G-B\right),a\left(2R-G-B\right)\right)$$

Assuming a>0, this is always verified, as both arguments to the arctan2 function are scaled by an equal amount.

*$\alpha_{R}$ component*:

(A5) $$\arccos\left(\frac{R+G+B}{\sqrt{3}\sqrt{R^{2}+G^{2}+B^{2}}}\right)=\arccos\left(\frac{aR+aG+aB}{\sqrt{3}\sqrt{{\left(aR\right)}^{2}+{\left(aG\right)}^{2}+{\left(aB\right)}^{2}}}\right)$$

Scalar *a* can be collected in both the numerator and denominator from the right part of the equation, leading to:

(A6) $$\arccos\left(\frac{R+G+B}{\sqrt{3}\sqrt{R^{2}+G^{2}+B^{2}}}\right)=\arccos\left(\frac{a(R+G+B)}{a\sqrt{3}\sqrt{R^{2}+G^{2}+B^{2}}}\right)$$

Assuming a≠0, the two *a* scalars cancel out, verifying the equation.

*$\alpha_{Z}$ component*:

(A7) $$\sqrt{R^{2}+G^{2}+B^{2}}=\sqrt{{\left(aR\right)}^{2}+{\left(aG\right)}^{2}+{\left(aB\right)}^{2}}$$

Collecting scalar *a* in the right part of the equation does not lead to any simplification. The equation is not verified, unless for the trivial cases of a=1, or R=G=B=0.

### Appendix A.2. Light Intensity Shift

Described by the expression:

(A8) $$\left(\begin{array}{c} R\\ G\\ B \end{array}\right)+\left(\begin{array}{c} o_{1}\\ o_{1}\\ o_{1} \end{array}\right)$$

*$\alpha_{A}$ component*:

(A9) $$\begin{array}{c} \arctan2\left(\sqrt{3}\left(G-B\right),2R-G-B\right)=\\ \arctan2\left(\sqrt{3}\left(\left(G+o_{1}\right)-\left(B+o_{1}\right)\right),2\left(R+o_{1}\right)-\left(G+o1\right)-\left(B+o_{1}\right)\right) \end{array}$$

Expanding the right part of the equation, we have:

(A10) $$\arctan2\left(\sqrt{3}\left(G+o_{1}-B-o_{1}\right),2R+2o_{1}-G-o1-B-o_{1}\right)$$

Terms $o_{1}$ cancel out in both arguments, thus always verifying Equation (A9).

*$\alpha_{R}$ component*:

(A11) $$\arccos\left(\frac{R+G+B}{\sqrt{3}\sqrt{R^{2}+G^{2}+B^{2}}}\right)=\arccos\left(\frac{R+o_{1}+G+o_{1}+B+o_{1}}{\sqrt{3}\sqrt{{\left(R+o_{1}\right)}^{2}+{\left(G+o_{1}\right)}^{2}+{\left(B+o_{1}\right)}^{2}}}\right)$$

The equation admits the following solution:

(A12) $$o_{1}=-\frac{2}{3}\left(R+G+B\right)$$

This would classify as a conditional invariance. However, it can be shown that the constraint from Equation (A12) leads to configurations that are not admissible in our domain, as the resulting negative $o_{1}$ is large enough that it would always produce negative values in either color channel. In other words the inequality:

(A13) $$\frac{2}{3}\left(R+G+B\right)<\min\left(R,G,B\right)$$

has no solutions for R>0, G>0 and B>0.

*$\alpha_{Z}$ component*:

(A14) $$\sqrt{R^{2}+G^{2}+B^{2}}=\sqrt{{\left(R+o_{1}\right)}^{2}+{\left(G+o_{1}\right)}^{2}+{\left(B+o_{1}\right)}^{2}}$$

The equation also admits a solution in the form:

(A15) $$o_{1}=-\frac{2}{3}\left(R+G+B\right)$$

By the same proof provided for Equation (A11), this has no practical solutions for R>0, G>0 and B>0.

### Appendix A.3. Light Intensity Change and Shift

Described by the transformation:

(A16) $$\left(\begin{array}{ccc} a & 0 & 0\\ 0 & a & 0\\ 0 & 0 & a \end{array}\right)\left(\begin{array}{c} R\\ G\\ B \end{array}\right)+\left(\begin{array}{c} o_{1}\\ o_{1}\\ o_{1} \end{array}\right)$$

*$\alpha_{A}$ component*:

(A17) $$\begin{array}{c} \arctan2\left(\sqrt{3}\left(G-B\right),2R-G-B\right)=\\ \arctan2\left(\sqrt{3}\left(\left(aG+o_{1}\right)-\left(aB+o_{1}\right)\right),2\left(aR+o_{1}\right)-\left(aG+o1\right)-\left(aB+o_{1}\right)\right) \end{array}$$

By combining the simplifications from Equations (A4) and (A10), both the offset component $o_{1}$ and the scalar component *a* cancel out, thus verifying the equation.

*$\alpha_{R}$ component*:

(A18) $$\arccos\left(\frac{R+G+B}{\sqrt{3}\sqrt{R^{2}+G^{2}+B^{2}}}\right)=\arccos\left(\frac{aR+o_{1}+aG+o_{1}+aB+o_{1}}{\sqrt{3}\sqrt{{\left(aR+o_{1}\right)}^{2}+{\left(aG+o_{1}\right)}^{2}+{\left(aB+o_{1}\right)}^{2}}}\right)$$

The scalar component *a* can be collected and simplified following Equation (A6). However, the offset $o_{1}$ leads to the same unfeasible conditional invariance from Equation (A12), effectively resulting in non-invariance.

*$\alpha_{Z}$ component*:

(A19) $$\sqrt{R^{2}+G^{2}+B^{2}}=\sqrt{{\left(aR+o_{1}\right)}^{2}+{\left(aG+o_{1}\right)}^{2}+{\left(aB+o_{1}\right)}^{2}}$$

The relationship can be expressed in terms of $o_{1}$, with the interpretation that an appropriate value of $o_{1}$ can be selected in order to compensate for the effect of the transformation introduced by scalar *a*:

(A20) $$o_{1}=\frac{1}{3}\left(\pm \sqrt{\left(3-2a^{2}\right)B^{2}+2a^{2}B\left(G+R\right)+\left(3-2a^{2}\right)G^{2}+2a^{2}GR+\left(3-2a^{2}\right)R^{2}}-a\left(B+G+R\right)\right)$$

This conditional invariance has no evident unfeasibility features (contrarily to the case for Equation (A12)).

### Appendix A.4. Light Color Change

Described by the transformation:

(A21) $$\left(\begin{array}{ccc} a & 0 & 0\\ 0 & b & 0\\ 0 & 0 & c \end{array}\right)\left(\begin{array}{c} R\\ G\\ B \end{array}\right)$$

*$\alpha_{A}$ component*:

(A22) $$\arctan2\left(\sqrt{3}\left(G-B\right),2R-G-B\right)=\arctan2\left(\sqrt{3}\left(bG-cB\right),2aR-bG-cB\right)$$

The equation offers the following solution for *c* as a function of the other scalars *a* and *b*, as well as the input RGB values:

(A23) $$c=\frac{-\frac{2\sqrt{3}aR\left(B-G\right)}{B+G-2R}+\frac{\sqrt{3}bG\left(B-G\right)}{B+G-2R}+\sqrt{3}bG}{\sqrt{3}B-\frac{\sqrt{3}B\left(B-G\right)}{B+G-2R}}$$

*$\alpha_{R}$ component*:

(A24) $$\arccos\left(\frac{R+G+B}{\sqrt{3}\sqrt{R^{2}+G^{2}+B^{2}}}\right)=\arccos\left(\frac{aR+bG+cB}{\sqrt{3}\sqrt{{\left(aR\right)}^{2}+{\left(bG\right)}^{2}+{\left(cB\right)}^{2}}}\right)$$

By re-arranging the terms and solving for *c*, this equation is proven to accept the following two solutions:

(A25) $$c=\frac{\pm \sqrt{B^{2}{\left(-2aR-2bG\right)}^{2}-4B^{2}\left(\frac{{\left(B+G+R\right)}^{2}}{B^{2}+G^{2}+R^{2}}-1\right)\left(\frac{a^{2}R^{2}{\left(B+G+R\right)}^{2}}{B^{2}+G^{2}+R^{2}}-a^{2}R^{2}-2abGR+\frac{b^{2}G^{2}{\left(B+G+R\right)}^{2}}{B^{2}+G^{2}+R^{2}}-b^{2}G^{2}\right)}-B\left(-2aR-2bG\right)}{2B^{2}\left(\frac{{\left(B+G+R\right)}^{2}}{B^{2}+G^{2}+R^{2}}-1\right)}$$

*$\alpha_{Z}$ component*:

(A26) $$\sqrt{R^{2}+G^{2}+B^{2}}=\sqrt{{\left(aR\right)}^{2}+{\left(bG\right)}^{2}+{\left(cB\right)}^{2}}$$

Solved for *c*, this yields two solutions in the form:

(A27) $$c=\pm \frac{\sqrt{-a^{2}R^{2}-b^{2}G^{2}+B^{2}+G^{2}+R^{2}}}{B}$$

Given that both the numerator and denominator are positive, the only solution which produces a positive *c*, and thus which is admissible in our domain, is:

(A28) $$c=\frac{\sqrt{-a^{2}R^{2}-b^{2}G^{2}+B^{2}+G^{2}+R^{2}}}{B}$$

### Appendix A.5. Light Color Change and Shift

Described by the transformation:

(A29) $$\left(\begin{array}{ccc} a & 0 & 0\\ 0 & b & 0\\ 0 & 0 & c \end{array}\right)\left(\begin{array}{c} R\\ G\\ B \end{array}\right)+\left(\begin{array}{c} o_{1}\\ o_{2}\\ o_{3} \end{array}\right),$$

*$\alpha_{A}$ component*:

(A30) $$\arctan2\left(\sqrt{3}\left(G-B\right),2R-G-B\right)=\arctan2\left(\sqrt{3}\left(bG-cB\right),2aR-bG-cB\right)$$

(A31) $$\arctan2\left(\sqrt{3}\left(G-B\right),2R-G-B\right)=\arctan2\left(\sqrt{3}\left(\left(G+o_{2}\right)-\left(B+o_{3}\right)\right),2\left(R+o_{1}\right)-\left(G+o_{2}\right)-\left(B+o_{3}\right)\right)$$

Equation (A30) produces the same solution from Appendix A.4:

(A32) $$c=\frac{-\frac{2\sqrt{3}aR\left(B-G\right)}{B+G-2R}+\frac{\sqrt{3}bG\left(B-G\right)}{B+G-2R}+\sqrt{3}bG}{\sqrt{3}B-\frac{\sqrt{3}B\left(B-G\right)}{B+G-2R}}$$

Equation (A31) yields an additional constraint:

(A33) $$o_{3}=\frac{-Bo_{1}+Bo_{2}+Go_{1}-o_{2}R}{G-R}$$

*$\alpha_{R}$ component*:

(A34) $$\arccos\left(\frac{R+G+B}{\sqrt{3}\sqrt{R^{2}+G^{2}+B^{2}}}\right)=\arccos\left(\frac{aR+bG+cB}{\sqrt{3}\sqrt{{\left(aR\right)}^{2}+{\left(bG\right)}^{2}+{\left(cB\right)}^{2}}}\right)$$

(A35) $$\arccos\left(\frac{R+G+B}{\sqrt{3}\sqrt{R^{2}+G^{2}+B^{2}}}\right)=\arccos\left(\frac{R+o_{1}+G+o_{2}+B+o_{3}}{\sqrt{3}\sqrt{{\left(R+o_{1}\right)}^{2}+{\left(G+o_{2}\right)}^{2}+{\left(B+o_{3}\right)}^{2}}}\right)$$

Equation (A34) produces the same solution from Appendix A.4:

(A36) $$c=\frac{\pm \sqrt{B^{2}{\left(-2aR-2bG\right)}^{2}-4B^{2}\left(\frac{{\left(B+G+R\right)}^{2}}{B^{2}+G^{2}+R^{2}}-1\right)\left(\frac{a^{2}R^{2}{\left(B+G+R\right)}^{2}}{B^{2}+G^{2}+R^{2}}-a^{2}R^{2}-2abGR+\frac{b^{2}G^{2}{\left(B+G+R\right)}^{2}}{B^{2}+G^{2}+R^{2}}-b^{2}G^{2}\right)}-B\left(-2aR-2bG\right)}{2B^{2}\left(\frac{{\left(B+G+R\right)}^{2}}{B^{2}+G^{2}+R^{2}}-1\right)}$$

Equation (A35) yields an additional constraint:

(A37) $$\begin{array}{c} o_{3}=\frac{1}{2(BG+BR+GR)}\\ (-\left(B^{2}G\right)+B^{2}o_{1}+B^{2}o_{2}-B^{2}R+\\ \pm \sqrt{}({\left(B^{2}G-B^{2}o_{1}-B^{2}o_{2}+B^{2}R+2BGR-G^{2}o_{1}-G^{2}o_{2}-G^{2}R-G^{3}-GR^{2}-o_{1}R^{2}-o_{2}R^{2}-R^{3}\right)}^{2}+\\ -4\left(BG+BR+GR\right)(-B^{2}Go_{1}-B^{2}o_{1}o_{2}+B^{3}\left(-o_{1}\right)-B^{2}o_{2}R-B^{3}o_{2}-BG^{2}o_{1}+BG^{2}o_{2}+BG{o_{1}}^{2}+\\ +2BGo_{1}R+BG{o_{2}}^{2}+2BGo_{2}R+B{o_{1}}^{2}R+Bo_{1}R^{2}+B{o_{2}}^{2}R-Bo_{2}R^{2}-G^{2}o_{1}o_{2}-G^{3}o_{1}+\\ +G^{2}o_{2}R+G{o_{1}}^{2}R+Go_{1}R^{2}+G{o_{2}}^{2}R-o_{1}o_{2}R^{2}-o_{2}R^{3}))+\\ -2BGR+G^{2}o_{1}+G^{2}o_{2}+G^{2}R+G^{3}+GR^{2}+o_{1}R^{2}+o_{2}R^{2}+R^{3}) \end{array}$$

*$\alpha_{Z}$ component*:

(A38) $$\sqrt{R^{2}+G^{2}+B^{2}}=\sqrt{{\left(aR\right)}^{2}+{\left(bG\right)}^{2}+{\left(cB\right)}^{2}}$$

(A39) $$\sqrt{R^{2}+G^{2}+B^{2}}=\sqrt{{\left(R+o_{1}\right)}^{2}+{\left(G+o_{2}\right)}^{2}+{\left(B+o_{2}\right)}^{2}}$$

Equation (A38) produces the same solution from Appendix A.4:

(A40) $$c=\frac{\sqrt{-a^{2}R^{2}-b^{2}G^{2}+B^{2}+G^{2}+R^{2}}}{B}$$

Equation (A39) yields an additional constraint:

(A41) $$o_{3}=\pm \sqrt{B^{2}-o_{2}\left(2G+o_{2}\right)-o_{1}^{2}-2o_{1}R}-B$$

Alternatively, Equations (A38) and (A39) can be rewritten as:

(A42) $$\sqrt{R^{2}+G^{2}+B^{2}}=\sqrt{{\left(aR+o_{1}\right)}^{2}+{\left(bG+o_{2}\right)}^{2}+{\left(cB+o_{2}\right)}^{2}}$$

By re-arranging the terms and solving for $o_{3}$, this equation accepts the following two solutions:

(A43) $$o_{3}=\pm \sqrt{-a^{2}R^{2}-2ao_{1}R-b^{2}G^{2}-2bGo_{2}+B^{2}+G^{2}-o_{1}^{2}-o_{2}^{2}+R^{2}}-Bc$$
